# Supplementary figures and images for: Suppression of HIV-TAT and cocaine-induced neurotoxicity and inflammation by cell penetrable itaconate esters
Source: J Neurovirol. 2024 Jun 17;30(4):337–52. doi: 10.1007/s13365-024-01216-9 (PMC11512888; doi:10.1007/s13365-024-01216-9)

# Supplemental Figure 1

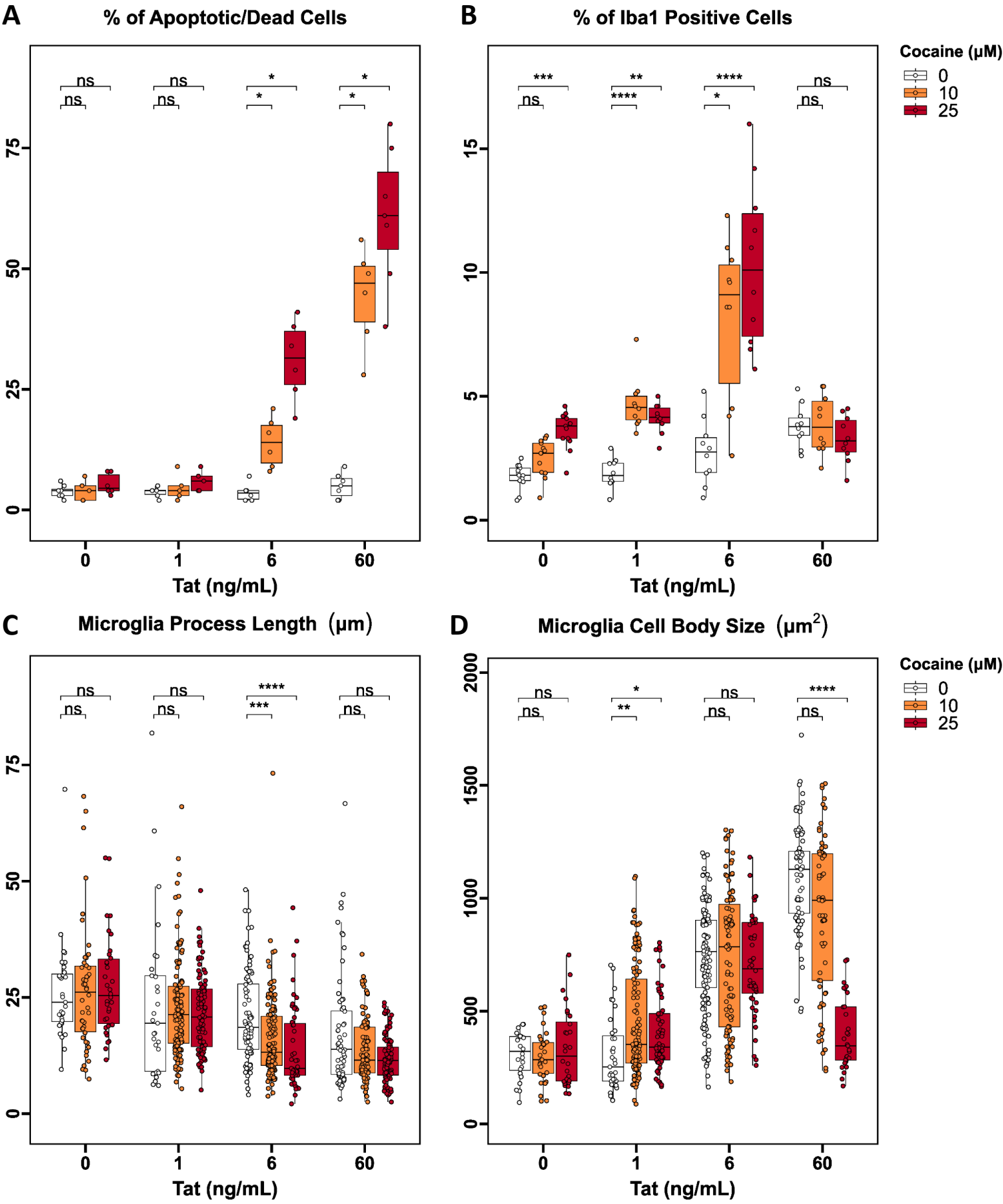

Supplement: Supplementary file 1 — Supplementary file1: Effects of Tat and/or cocaine on cell viability and microglia morphology in primary cortical cultures (a) Boxplots showing the percentage of dead/apoptotic cells in primary cortical cultures treated with Tat (1, 6, or 60 ng/mL) for 48 h and/or cocaine (10 or 25 μM) for an additional 24 h. The x-axis represents increasing Tat concentration, and the colors indicate different cocaine concentrations (Orange: 10 μM; Red: 25 μM). (b) Percentage of Iba1-positive microglia cells in each experimental group. Each data point corresponds to an image containing 300-450 cells. (c) Quantification of the length of microglia processes. (d) Measurement of microglia cell body size. Each box covers 50% of the data, and the line inside indicates the median. Statistical significance was determined using the Mann-Whitney-Wilcoxon test, followed by Bonferroni post hoc test (ns, not significant; *, p<0.05; **, p<0.01; ***, P<0.001) (a) Boxplots showing the percentage of dead/apoptotic cells in primary cortical cultures treated with Tat (1, 6, or 60 ng/mL) for 48 h and/or cocaine (10 or 25 μM) for an additional 24 h. The x-axis represents increasing Tat concentration, and the colors indicate different cocaine concentrations (Orange: 10 μM; Red: 25 μM) (b) Percentage of Iba1-positive microglia cells in each experimental group. Each data point corresponds to an image containing 300-450 cells (c) Quantification of the length of microglia processes (d) Measurement of microglia cell body size. Each box covers 50% of the data, and the line inside indicates the median. Statistical significance was determined using the Mann-Whitney-Wilcoxon test, followed by Bonferroni post hoc test (ns, not significant; *, p<0.05; **, p<0.01; ***, P<0.001) (PDF 425 KB) [file 13365_2024_1216_MOESM1_ESM.pdf]

# Supplemental Figure 2

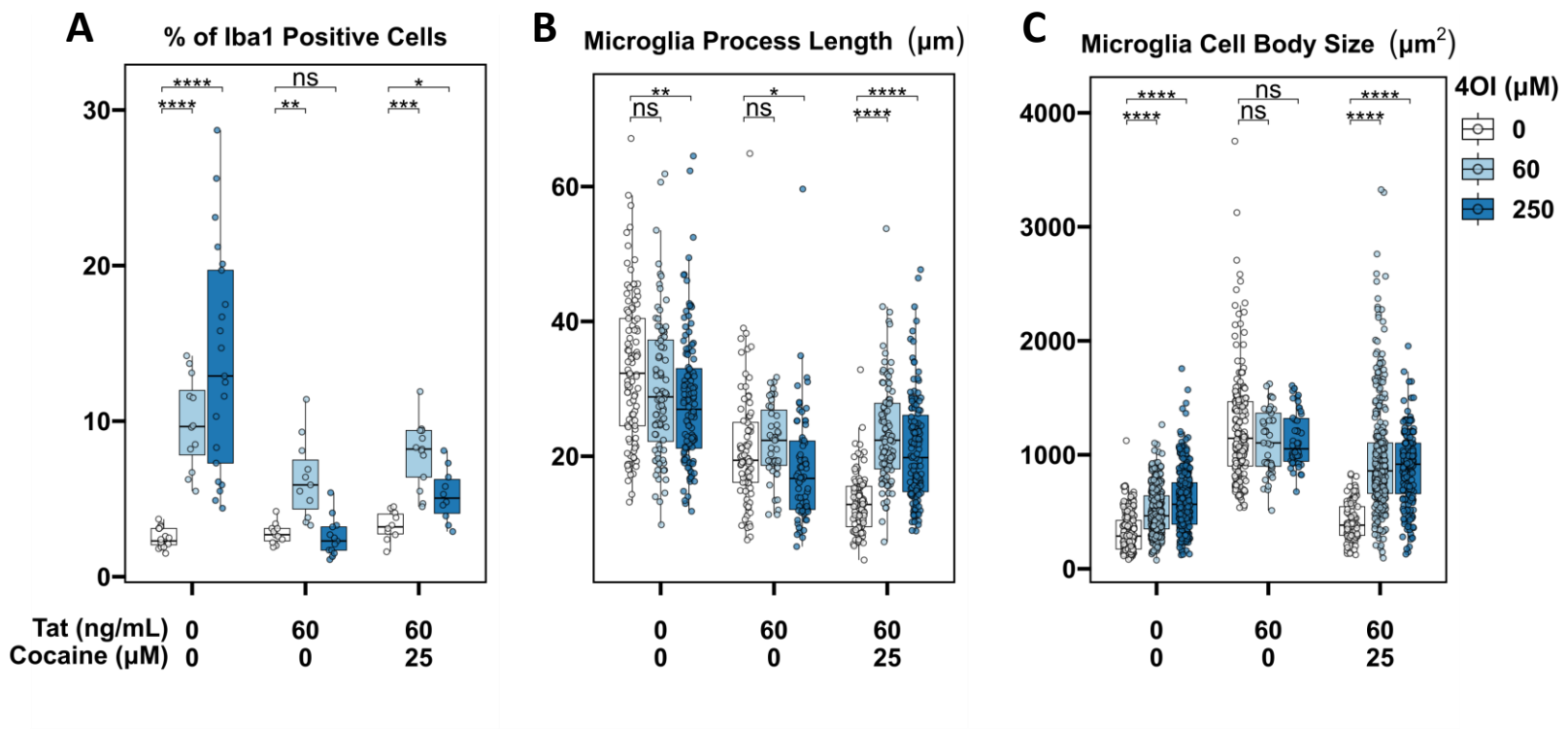

Supplement: Supplementary file 2 — Supplementary file2: Effects of Tat and cocaine on microglia cell morphology in primary cortical cultures (a) Proportion of Iba1 positive microglial cells in cultures treated with high Tat (60 ng/mL) and/or 4OI (60 or 250 µM) for 48 h, followed by an additional 24 h with or without cocaine (25 µM) (b) Length of microglia processes and (C) microglia cell body size in the same cultures. The intensity of the blue color indicates increasing 4OI concentration (60, 250 µM). The box covers 50% of the data in each condition, and the line inside indicates the median. Each point corresponds to an image containing 300-450 cells. The Mann-Whitney-Wilcoxon test was conducted to calculate the statistical significance, followed by Bonferroni post hoc test (ns, not significant; *, p<0.05; **, p<0.01; ***, P<0.001) (PDF 320 KB) [file 13365_2024_1216_MOESM2_ESM.pdf]

# Supplemental Figure 3

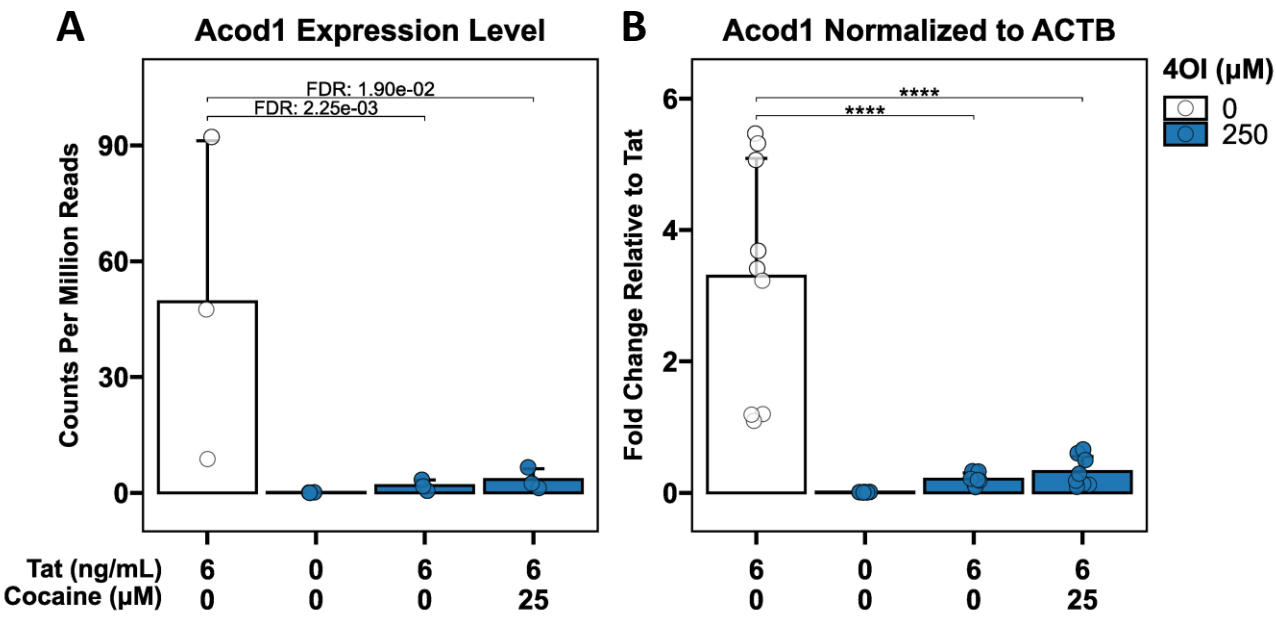

Supplement: Supplementary file 3 — Supplementary file3: The effect of 4OI treatment on Acod1 expression levels upon Tat or Tat-Cocaine treatment (a) Acod1 expression level, normalized to counts per million reads, after 48h of Tat treatment, with or without 4OI, and with or without cocaine exposure for 24 h (b) qPCR analysis demonstrating the fold change of Acod1, normalized to ACTB, following 48 h Tat, with or without 4OI, and with or without an additional cocaine exposure for 24 h (PDF 148 KB) [file 13365_2024_1216_MOESM3_ESM.pdf]

# Supplemental Figure 4

## GO: Biological Process

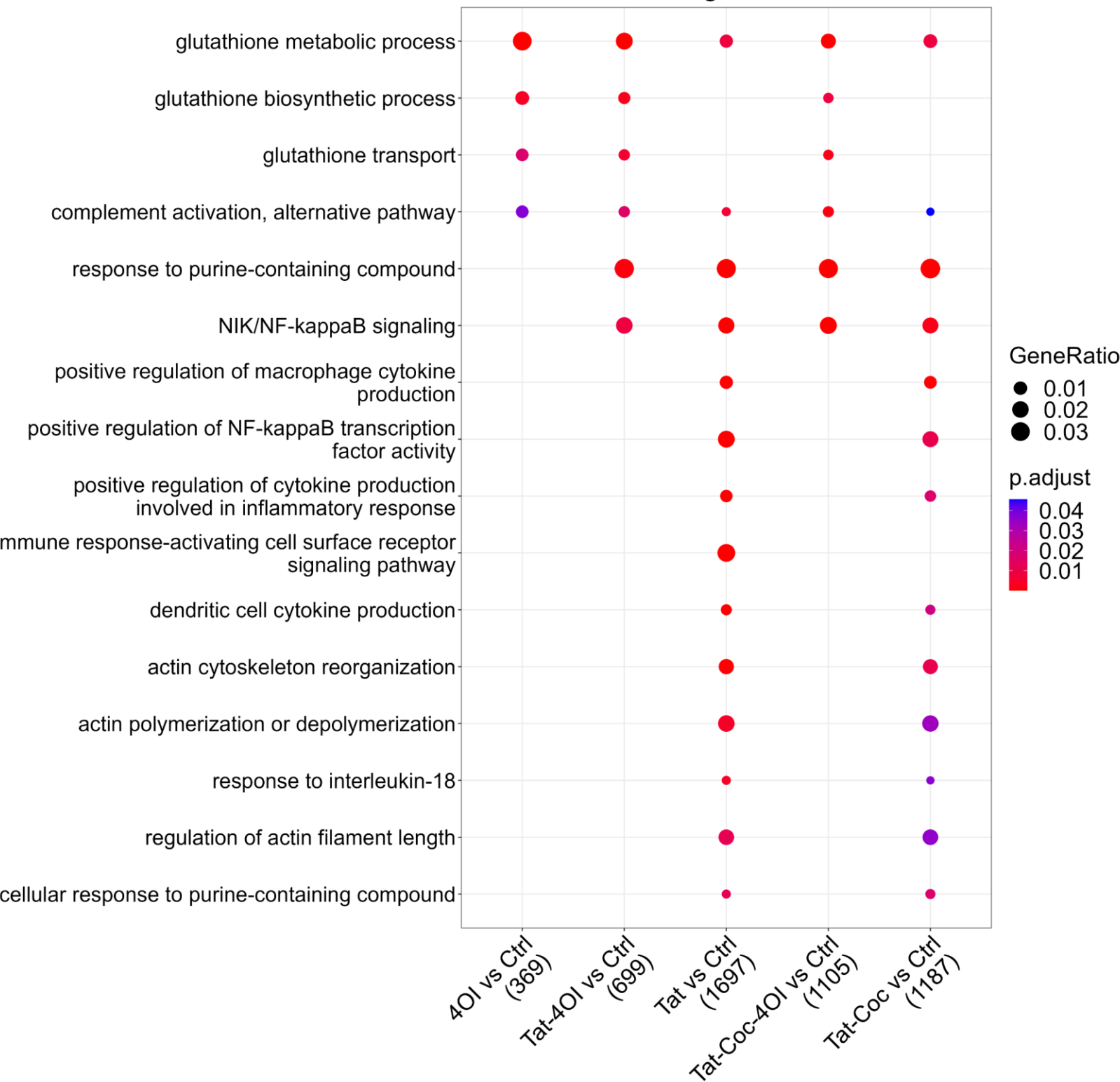

Supplement: Supplementary file 4 — Supplementary file4: The effect of 4OI treatment on transcriptomic profiles and pathways upon Tat or Tat-Cocaine treatment Dot plot of gene enrichment analyses of the differentially expressed genes in cells treated with Tat with or without 4OI for 48 h, with or without an additional 24 h of cocaine exposure. Gene ontology terms are labeled with name, sorted according to adjusted p-value and gene ratio (PDF 360 KB) [file 13365_2024_1216_MOESM4_ESM.pdf]
